# Supplementary material for: Delivered complementation in planta (DCIP) enables measurement of peptide-mediated protein delivery efficiency in plants
Source: Commun Biol. 2023 Aug 12;6:840. doi: 10.1038/s42003-023-05191-5 (PMC10423278; doi:10.1038/s42003-023-05191-5)
Supplement: Supplementary file 2 — Supplemental Figures [file 42003_2023_5191_MOESM2_ESM.pdf]

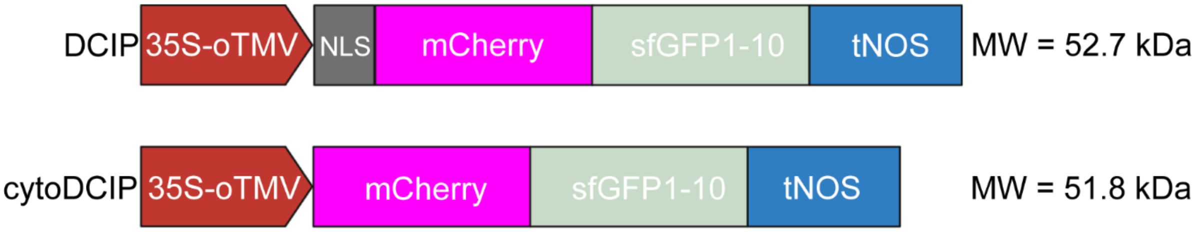

Supplemental Figure S1. Schematic of DCIP and cytoDCIP transcriptional unit (created with BioRender.com). Expression is driven by a 35s promoter and terminated by tNOS. DCIP possess a SV40 NLS for nuclear localization whereas cytoDCIP does not and localizes to the cytosol. Both vectors were constructed as level-1 assemblies in Goldenbraid 2.0.

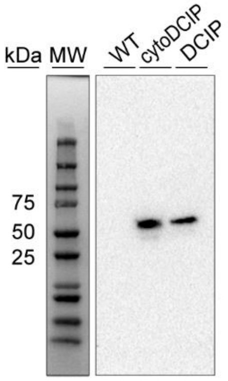

Supplemental Figure S2. Western Blot using anti-mCherry primary antibody and *N. benthamiana* leaf lysates 3 d.p.i. with either DCIP or cytoDCIP showing both protein fusions at the predicted molecular weight.

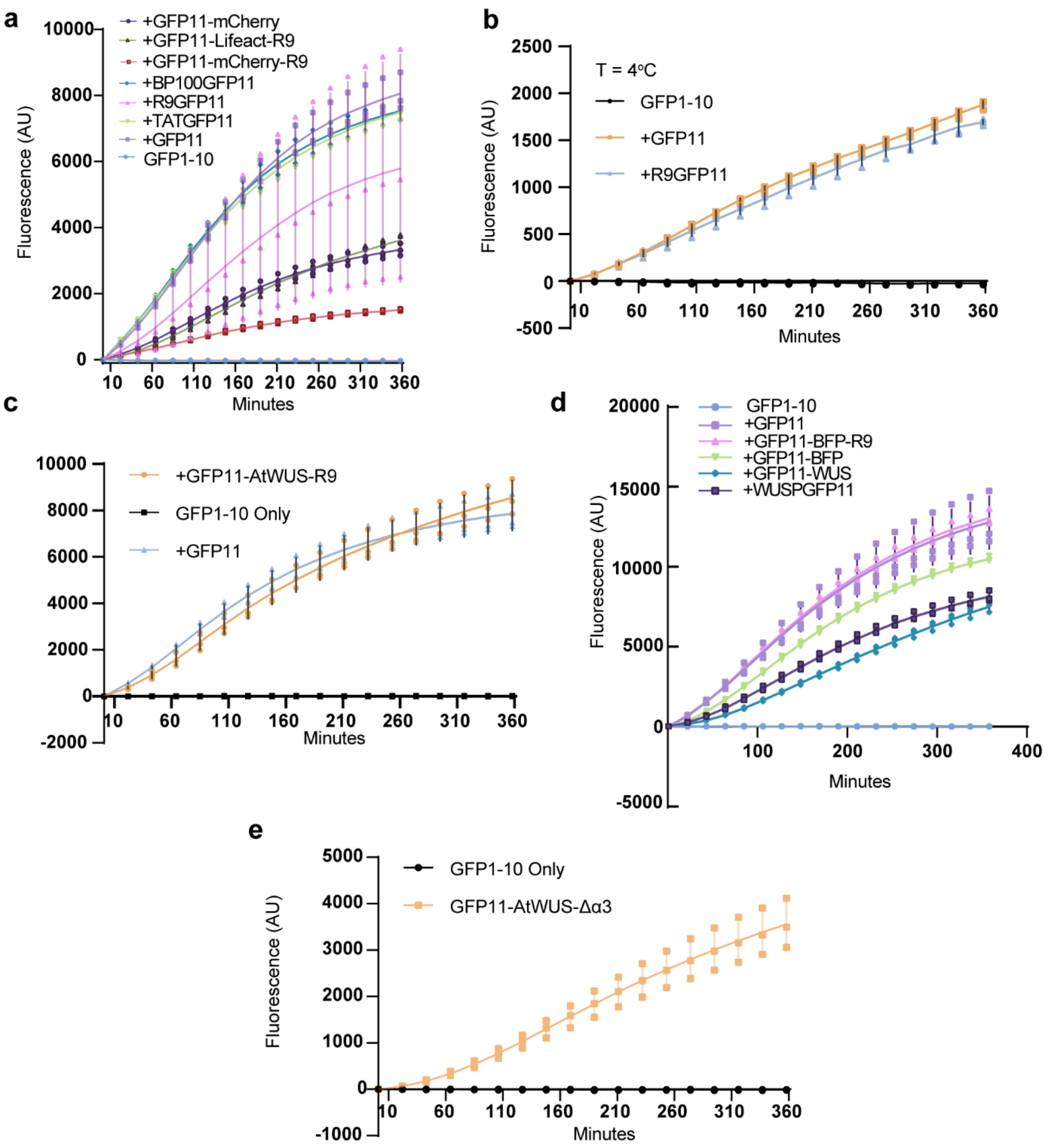

Supplemental Figure S3. a, *In vitro* GFP complementation assay using recombinantly expressed and purified sfGFP1-10 and GFP11 containing constructs used in this study. A final concentration of 5μM GFP1-10 his-tag eluate and 10μM of GFP11 containing protein or peptide were incubated for up to 6 hours. Fluorescence was measured every minute on a Biorad CFX96 qPCR machine set at 21°C or, **b**, 4°C. **c**, GFP complementation experiment with GFP11-AtWUS-R9. A final concentration of 6μM test protein and 4μM GFP1-10 in addition to 200mM Tris, pH 8.0 was used for this assay. **d**, GFP complementation experiment using same conditions as **a** but with proteins and peptides used later in this study. **e**, GFP complementation experiment using the same

conditions as panel A using the helix-3 deletion mutant, GFP11-AtWUS-Δα3. Plotted line is curve fit to mean of triplicate wells. Error bars are standard deviation of three triplicate wells. Only data from every 20 minutes of measurements are shown for clarity.

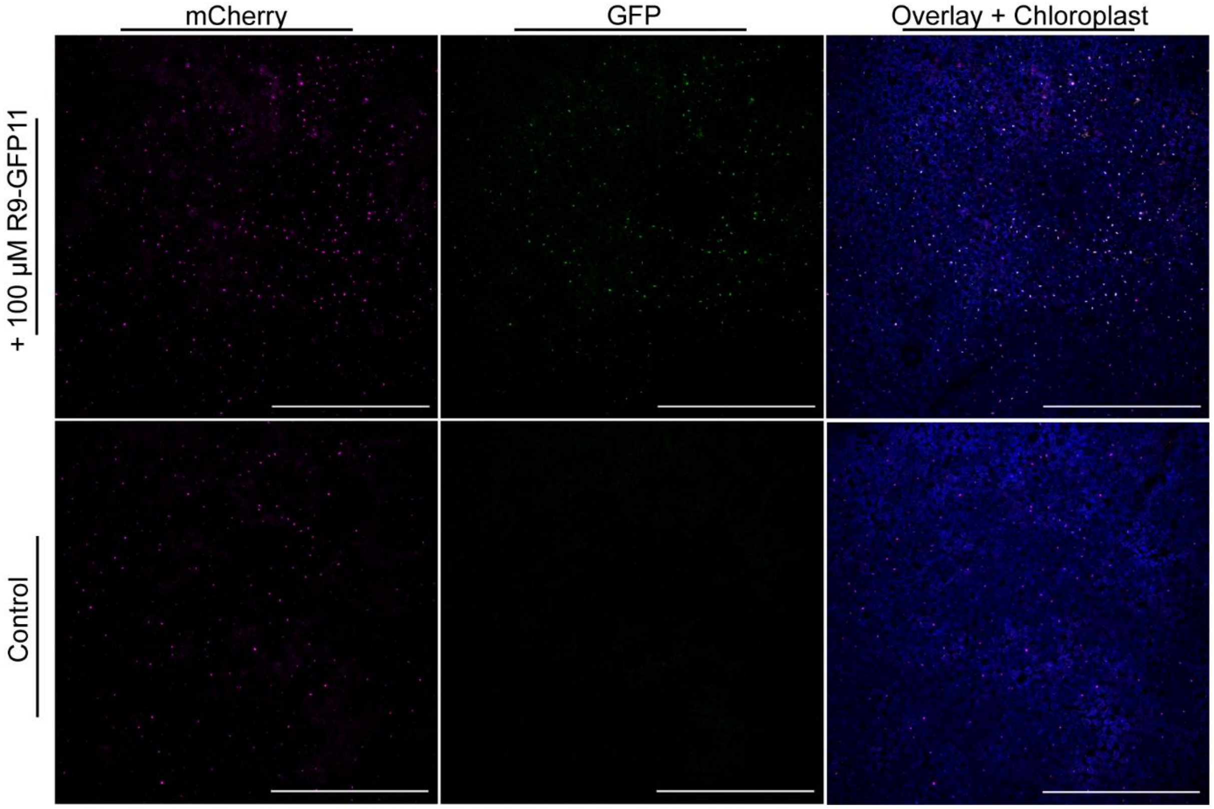

Supplemental Figure S4. DCIP expressing plant infiltrated with either 100μM R9-GFP11 or water control and imaged using confocal microscopy at 4-5H using a 5x objective. mCherry fluorescence is pseudocolored magenta and sfGFP fluorescence is colored green. Chloroplast autofluorescence is colored blue. Scale bar is 1mm.

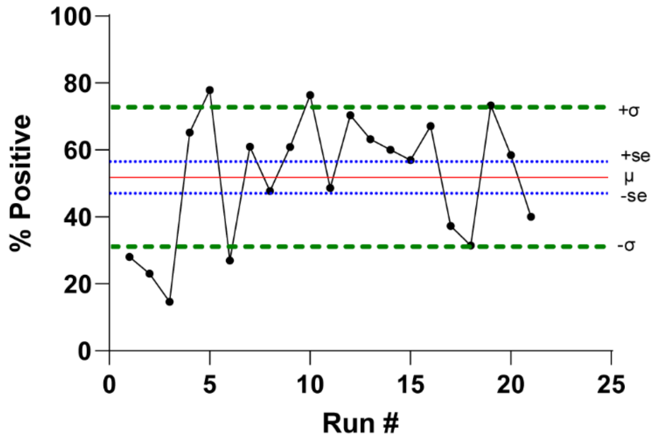

Supplemental Figure S5. Percentage of GFP positive nuclei in DCIP expressing *N. benthamiana* treated with 100μM R9-GFP11 for 4-5H in the first 21 DCIP experiments performed. Data were pooled across several experiments to probe the innate variability in R9 delivery across multiple plants. Standard error and standard deviation are marked with a dotted and dashed line respectively. The mean, 52%, is marked with a solid line.

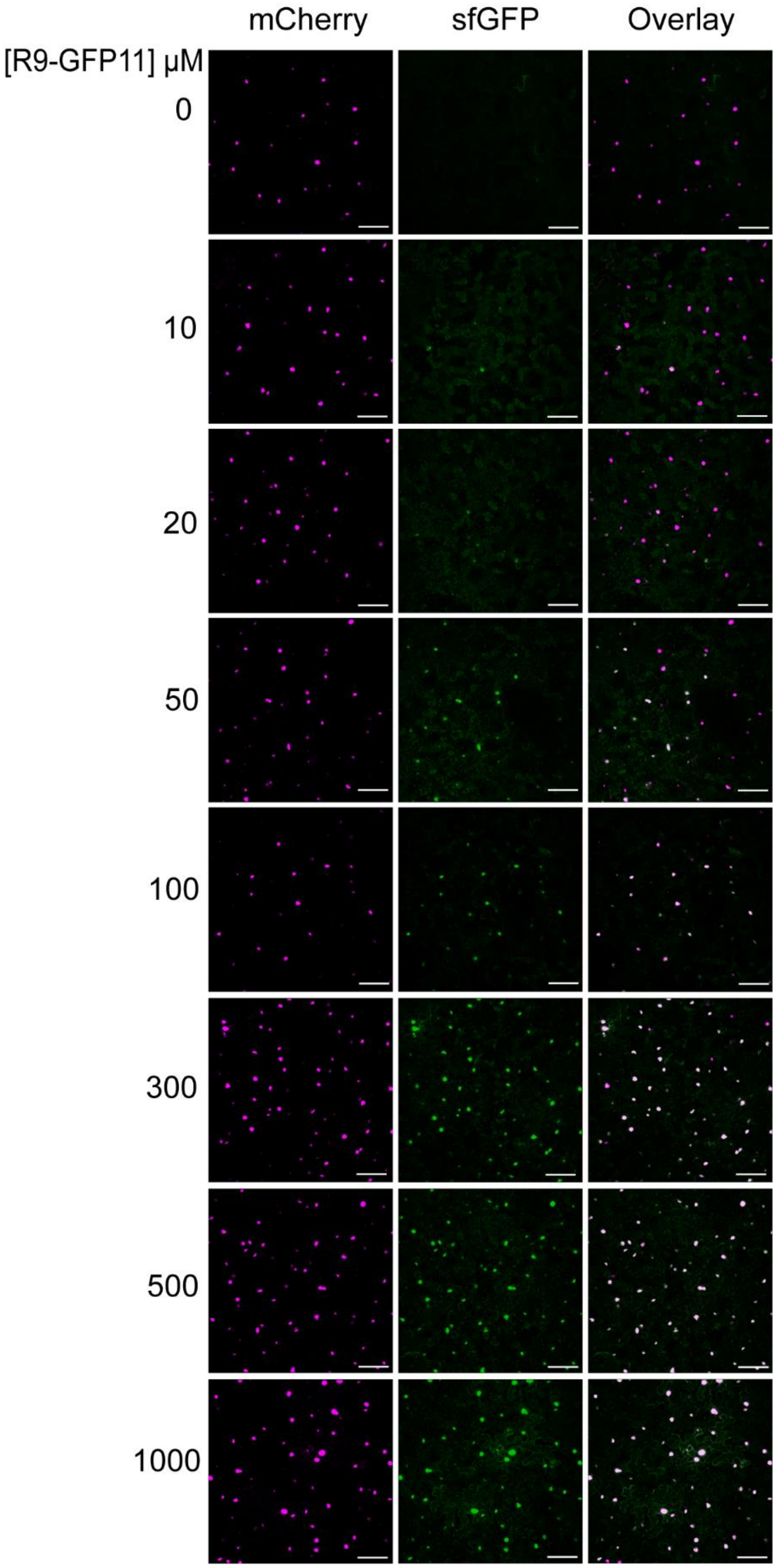

Supplemental Figure S6. Representative two-color maximum intensity projections of DCIP expressing leaves infiltrated with 0-1000 µM R9-GFP11 and incubated for 4-5 hours. Scale bar is 100µm. mCherry is pseudocolored magenta and sfGFP is pseudocolored green. Overlay results in white coloration

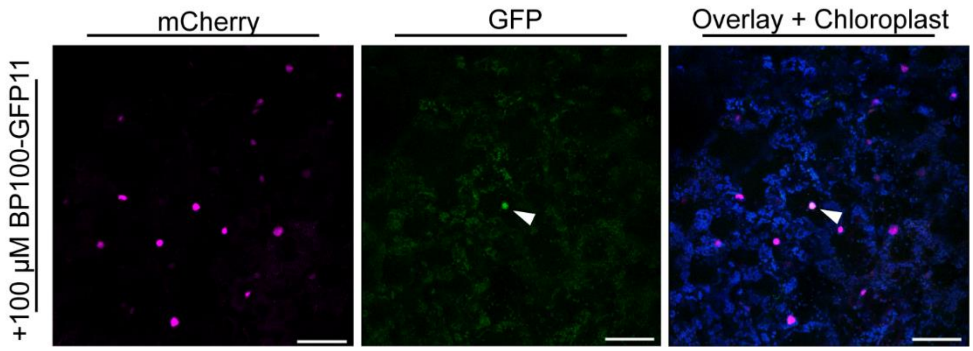

Supplemental Figure S7. Example maximum intensity projection of sfGFP complementation as resulting from 100µM BP100-GFP11 incubation in a DCIP expressing leaf disc for 4-5H. Scale bar is 100µm. sfGFP fluorescent nucleus is marked by a white triangle. mCherry is pseudocolored magenta, sfGFP is pseudocolored green, and chloroplast autofluorescence is pseudocolored blue. Overlay results in white coloration.

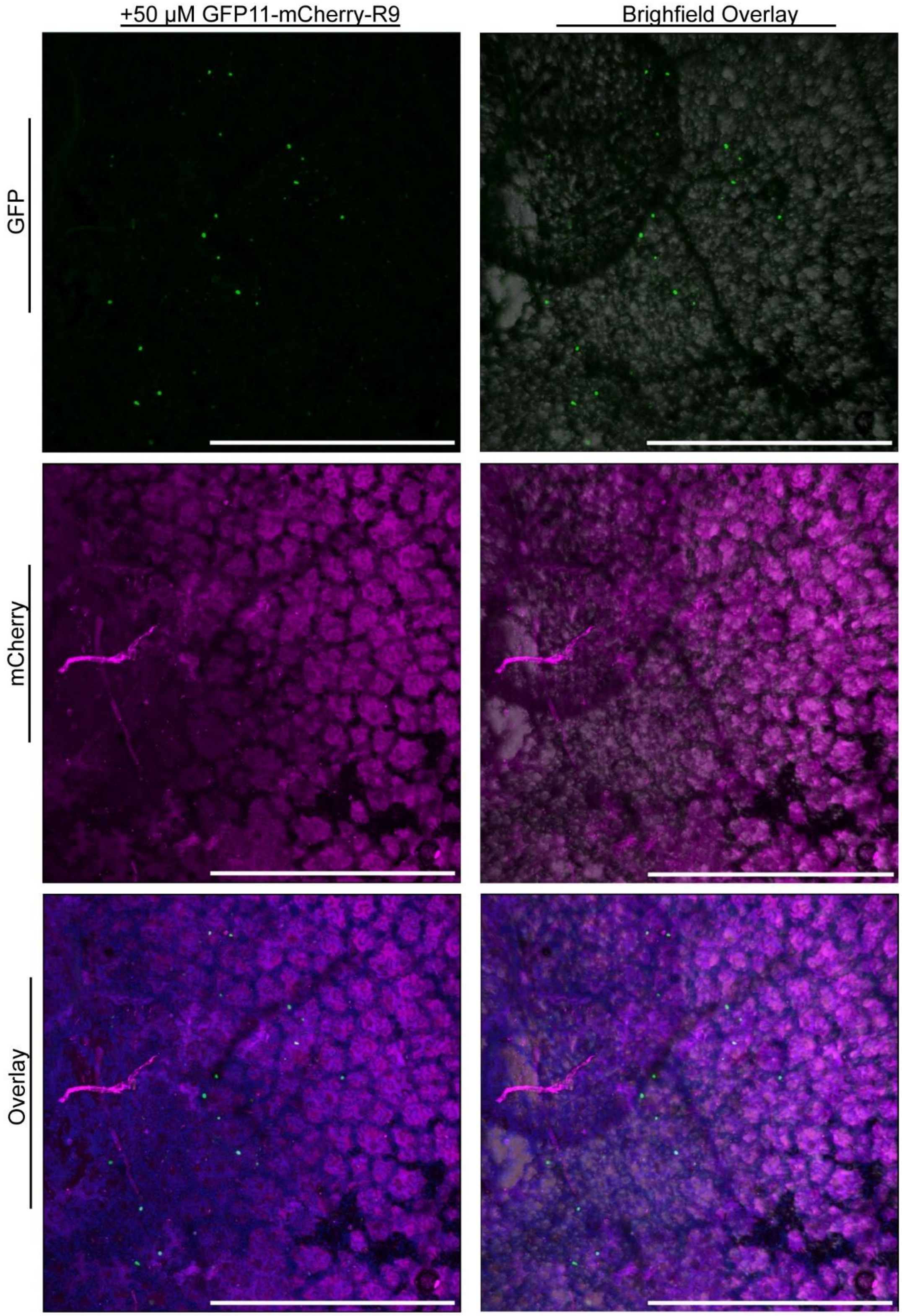

Supplemental Figure S8. DCIP expressing plant infiltrated with 50µM GFP11-mCherry-R9 and imaged using confocal microscopy at 5H using a 5x objective. mCherry fluorescence is pseudocolored magenta and sfGFP fluorescence is colored green. Green

nuclear fluorescence resulting from successful delivery appear as small circular objects. Chloroplast autofluorescence is colored blue. Transmitted light is overlayed to show anatomy. Scale bar is 1mm.

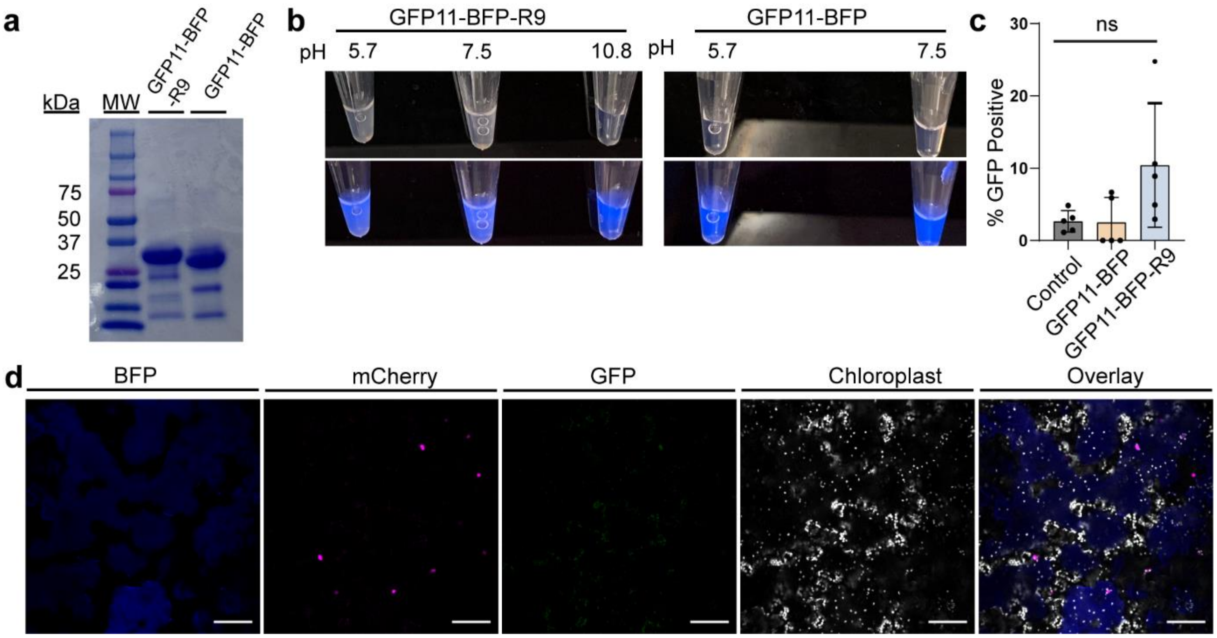

Supplemental Figure S9. **a**, SDS-PAGE gel of GFP11-BFP with or without R9 CPP tag. **b**, GFP11-BFP proteins at pH 5.7 50mM MES, pH 7.5 50mM Tris, or pH 10.8 50mM CAPS under white light or UV illumination. **c**, DCIP quantification for plants infiltrated with 100 µM starting protein of GFP11-BFP or GFP11-BFP-R9 at pH 7.5 and pH 9.0 respectively (N=5) and imaged at 6 hours post infiltration. Kruskal-Wallis test followed by Dunn's multiple comparisons test was performed where ns = p>0.05. Error bars represent standard deviation of the repeats. **d**, Representative single slice confocal micrographs of 100µM GFP11-BFP infiltrated DCIP expressing plant. BFP, mCherry, GFP, and Chloroplast are pseudocolored blue, magenta, green, and white respectively. Scalebar is 100 µm.

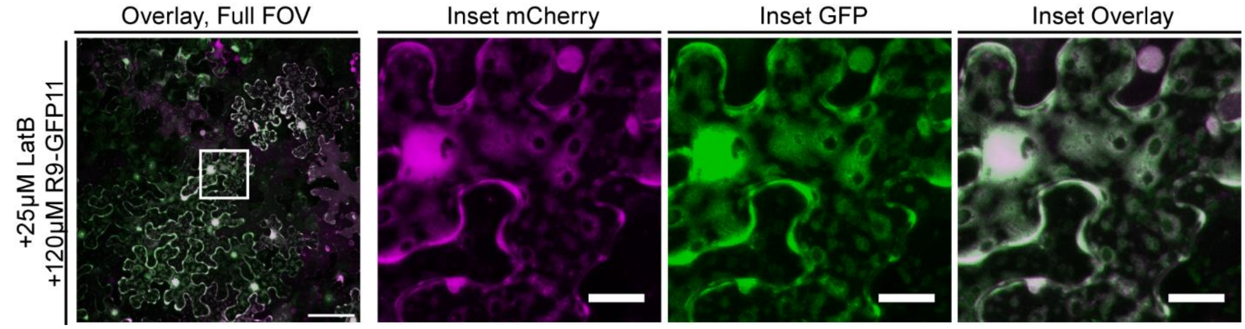

Supplemental Figure S10. Representative confocal micrograph (standard deviation projection) of a leaf disc from a cytoDCIP expressing leaf infiltrated with 120 µM R9-GFP11 and 25µM LatB after 6H of incubation. Full FOV scale bar is 100 µm; inset is 20 µm. mCherry is pseudocolored magenta and sfGFP pseudocolored green.

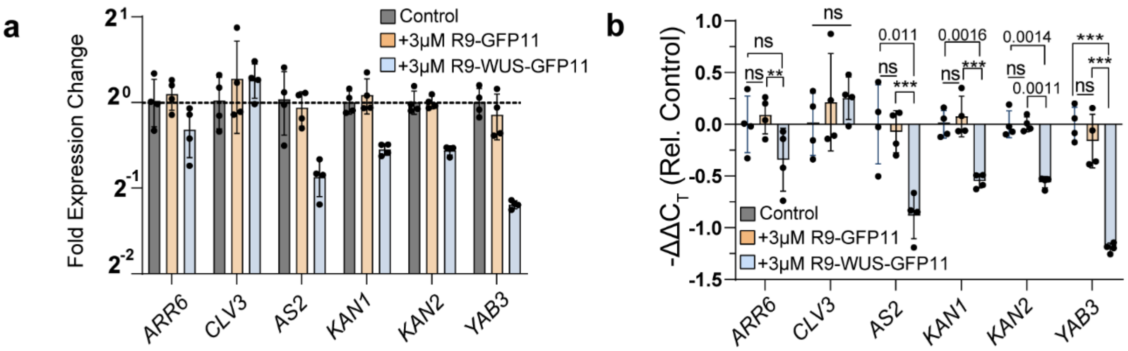

Supplemental Figure S11. a, Second repeat of GFP11-AtWUS-R9 treated seedlings rt-qPCR experiment using a separately purified batch of protein. Rt-qPCR analysis of downstream AtWUS genes in 12-day old *Arabidopsis* seedlings treated with 3 µM GFP11-AtWUS-R9 or R9-GFP11 for 24H. 8-10 seedlings were treated per well and four wells were utilized for each treatment (N=4). **b**, statistical comparison showing measured $\Delta\Delta C_T$ values of GFP11-AtWUS-R9 treated seedlings are significantly changed when compared to either R9-GFP11 treatment or no treatment. Statistical analysis performed with T-test comparison and Holm-Šídák correction for multiple comparisons where ns = p>0.05, exact p-values are given for 0.001<p<0.05, and *** = p< 0.001. Error bars represent standard deviation of the biological replicates.

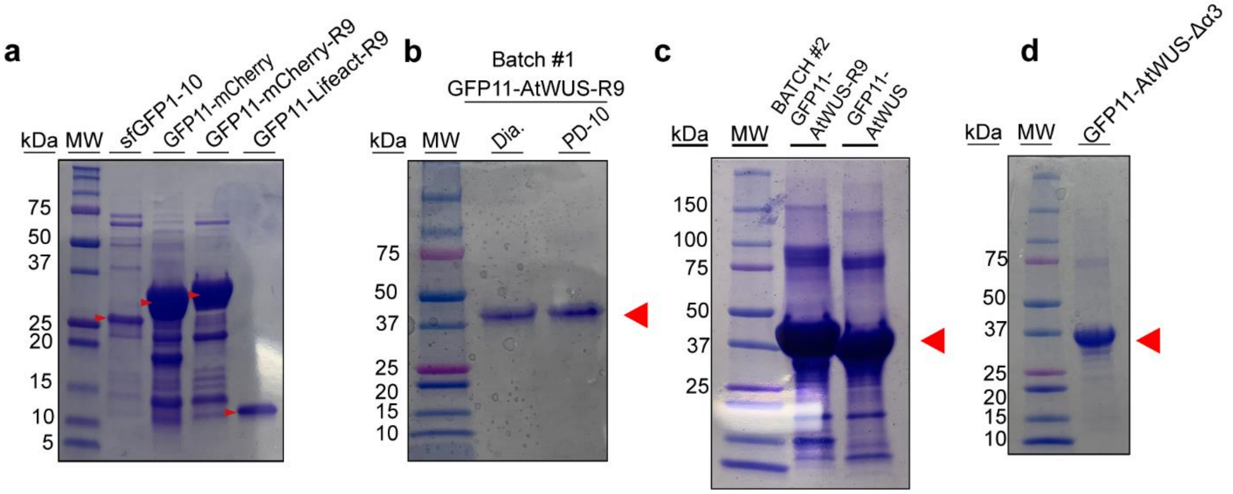

Supplemental Figure S12. a, SDS-PAGE of first four recombinant proteins used in this study. Red triangles indicate the protein of interest. Proteins were stained with Coomassie R-250. **b**, SDS-PAGE of purified GFP11-AtWUS-R9 exchanged into buffer P by dialysis or by desalting column. **c**, SDS-PAGE of purified GFP11-AtWUS-R9 (batch #2) and GFP11-AtWUS with no CPP. **d**, SDS-PAGE of the helix-3 deletion WUS mutant, GFP11-AtWUS-Δα3

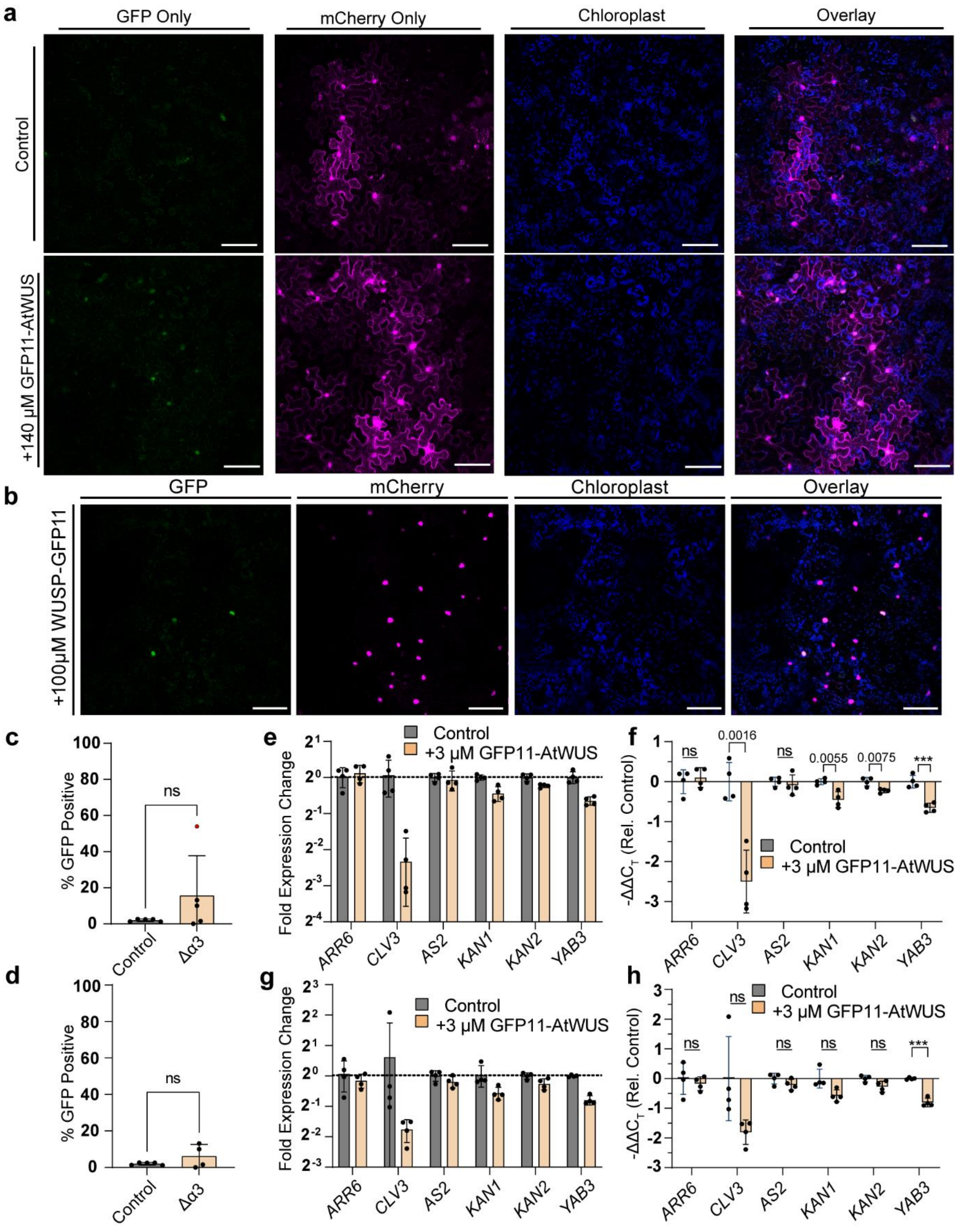

Supplemental Figure S13. a, representative maximum intensity projection confocal micrograph of a cytoDCIP expressing plant infiltrated with either buffer or 140 μM GFP11-AtWUS for 6 hours. Successful delivery and native nuclear-localization is indicated by

presence of nuclear-localized sfGFP fluorescence (pseudcolored green) in contrast to mCherry (magenta) being localized everywhere within the cell. Chloroplast are pseudocolored blue. Scalebar is 100 µm. **b**, maximum intensity projection of DCIP expressing plant infiltrated with 100 µM WUSP-GFP11 and imaged at 4-5 hours post infiltration. Successful delivery is indicated by presence of nuclear-localized sfGFP fluorescence (pseudcolored green). mCherry and chloroplast autofluorescence are pseudocolroed magenta and blue respectively. Scalebar is 100 µm. **c**, DCIP quantification for plants infiltrated with 140 µM GFP11-AtWUS-Δα3 (Δα3) (N=5) and imaged at 6 hours post infiltration. Red highlighted point identified as an outlier using Grubb's outlier test (α = 0.05). Statistical analysis was done using an unpaired T-test with p > 0.05 = ns. **d**, Data from panel C replotted without outlier with newly calculated mean and statistical comparison. Error bars represent standard deviation of the biological repeats. **e**, Rt-qPCR analysis of downstream AtWUS genes in 12-day old *Arabidopsis* seedlings treated with 3 µM GFP11-AtWUS for 24H. 8-10 seedlings were treated per well and four wells were utilized for each treatment (N=4). **f**, statistical comparison showing measured $\Delta\Delta C_T$ values of GFP11-AtWUS treated seedlings compared to the buffer control. Statistical analysis performed with T-test comparison controlled for false discovery rate using the method by Benjamini, Krieger, and Yekutieli where ns = p>0.05, , exact p-values are given for 0.001<p<0.05, and *** = p< 0.001. **g** and **h**, repeat of experiment displayed in panels **e** and **f**. For qPCR data, error bars represent standard deviation of the biological replicates.

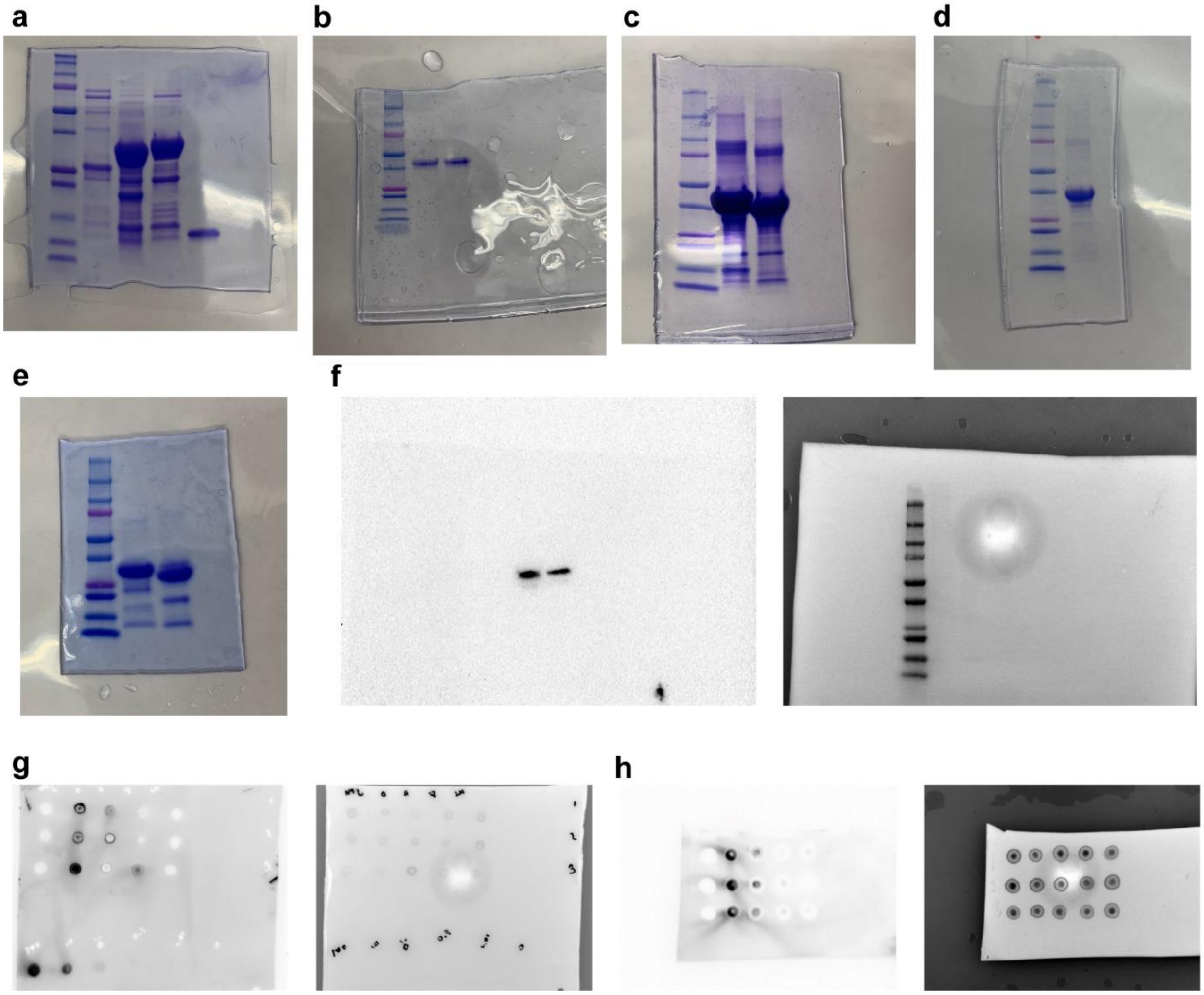

Supplemental Figure S14. Uncropped gel and blot images. **a,** Supplemental Figure S12a. **b,** Supplemental Figure S12b. **c,** Supplemental Figure S12c. **d,** Supplemental Figure S12d. **e,** Supplemental Figure S9a. **f,** Supplemental Figure S2 with accompanying white light photograph. **g,** Figure S2c with accompanying white light photograph. **h,** Second blot of Figure S2c with accompanying white light photograph.
