# Supplementary material for: Delivered complementation in planta (DCIP) enables measurement of peptide-mediated protein delivery efficiency in plants
Source: Commun Biol. 2023 Aug 12;6:840. doi: 10.1038/s42003-023-05191-5 (PMC10423278; doi:10.1038/s42003-023-05191-5)
Supplement: Supplementary file 3 — Description of Additional Supplementary Files [file 42003_2023_5191_MOESM3_ESM.pdf]

Description of Additional Supplementary Files

File name: Supplemental Data 1
Description: Table containing sequence data for generated plasmid constructs.

File name: Supplemental Data 2
Description: Table containing oligonucleotide sequence data used in the manuscript.
