# Supplementary material for: Delivered complementation in planta (DCIP) enables measurement of peptide-mediated protein delivery efficiency in plants
Source: Commun Biol. 2023 Aug 12;6:840. doi: 10.1038/s42003-023-05191-5 (PMC10423278; doi:10.1038/s42003-023-05191-5)
Supplement: Supplementary file 6 — Reporting Summary [file 42003_2023_5191_MOESM6_ESM.pdf]

nature portfolio

Corresponding author(s): Markita Landry, landry@berkeley.edu

Last updated by author(s): Jul 15, 2023

Reporting Summary

Nature Portfolio wishes to improve the reproducibility of the work that we publish. This form provides structure for consistency and transparency in reporting. For further information on Nature Portfolio policies, see our Editorial Policies and the Editorial Policy Checklist.

Statistics

For all statistical analyses, confirm that the following items are present in the figure legend, table legend, main text, or Methods section.

n/a	Confirmed	
☐	☒	The exact sample size (*n*) for each experimental group/condition, given as a discrete number and unit of measurement
☐	☒	A statement on whether measurements were taken from distinct samples or whether the same sample was measured repeatedly
☐	☒	The statistical test(s) used AND whether they are one- or two-sided<br>*Only common tests should be described solely by name; describe more complex techniques in the Methods section.*
☐	☒	A description of all covariates tested
☐	☒	A description of any assumptions or corrections, such as tests of normality and adjustment for multiple comparisons
☐	☒	A full description of the statistical parameters including central tendency (e.g. means) or other basic estimates (e.g. regression coefficient) AND variation (e.g. standard deviation) or associated estimates of uncertainty (e.g. confidence intervals)
☐	☒	For null hypothesis testing, the test statistic (e.g. *F*, *t*, *r*) with confidence intervals, effect sizes, degrees of freedom and *P* value noted<br>*Give P values as exact values whenever suitable.*
☒	☐	For Bayesian analysis, information on the choice of priors and Markov chain Monte Carlo settings
☒	☐	For hierarchical and complex designs, identification of the appropriate level for tests and full reporting of outcomes
☒	☐	Estimates of effect sizes (e.g. Cohen's *d*, Pearson's *r*), indicating how they were calculated

Our web collection on statistics for biologists contains articles on many of the points above.

Software and code

Policy information about availability of computer code

Data collection	Imaging data were collected on a Zeiss LSM880 laser scanning confocal microscope running Zeiss ZEN Black. GFP complementation data were collected on a Biorad CFX96 qPCR machine running Maestro ver. 4.1
Data analysis	Z-stack Images were preprocessed in ImageJ ver. 1.53q before being analyzed using CellProfiler 3.0 using the built-in Otsu's method for thresholding and image segmentation. Resulting data in CSVs were analyzed using a custom Python script written in Python 3.9. The script was used to develop statistical cutoffs for GFP positive cells and preparation of plotting in GraphPad Prism 9. Written code is publicly available at: 10.5281/zenodo.7272340. All statistical analysis were conducted using GraphPad Prism 9 using in-built methods as described in the manuscript text.

For manuscripts utilizing custom algorithms or software that are central to the research but not yet described in published literature, software must be made available to editors and reviewers. We strongly encourage code deposition in a community repository (e.g. GitHub). See the Nature Portfolio guidelines for submitting code & software for further information.

Data

Policy information about availability of data

All manuscripts must include a data availability statement. This statement should provide the following information, where applicable:

- Accession codes, unique identifiers, or web links for publicly available datasets
- A description of any restrictions on data availability
- For clinical datasets or third party data, please ensure that the statement adheres to our policy

The code from this study are available in the Zenodo repository with the identifier: 10.5281/zenodo.7272340. Plasmids generated for this study are available through Addgene (#193860-193867 and #202053-202056) upon final release. All source data, including images, related to this study are available from Dryad with the identifier: https://doi.org/10.6078/D1ZB1S. All raw, uncropped, blot and gel images are available in Supplemental Figure S14 and also the Dryad repository. Further correspondence and requests for materials should be addressed to M.P.L.

Human research participants

Policy information about studies involving human research participants and Sex and Gender in Research.

Reporting on sex and gender	N/A
Population characteristics	N/A
Recruitment	N/A
Ethics oversight	N/A

Note that full information on the approval of the study protocol must also be provided in the manuscript.

Field-specific reporting

Please select the one below that is the best fit for your research. If you are not sure, read the appropriate sections before making your selection.

☒ Life sciences ☐ Behavioural & social sciences ☐ Ecological, evolutionary & environmental sciences

For a reference copy of the document with all sections, see nature.com/documents/nr-reporting-summary-flat.pdf

Life sciences study design

All studies must disclose on these points even when the disclosure is negative.

Sample size	Sample-size calculations were not performed. A sample size of at least 3 biological replicates, with each replicate being a different plant, was taken in all cases.
Data exclusions	No data were excluded.
Replication	All experiments were performed independently at least two times using new individual plants to account for plant-to-plant variation. Attempts at replication were successful. For WUSCHEL-R9 transcription factor delivery, two separate batches of proteins were used to confirm repeatability.
Randomization	Samples were imaged in random order when possible.
Blinding	Blinding was not relevant to this study as the goal was to demonstrate the capabilities of a newly developed technique for detecting cytosolic protein delivery in plants. Thus, the individual identity of samples was not important to the outcome of the study. In the case of imaging, the control sample needed to be labeled ahead of time to act as a baseline for determining positive delivery events.

Reporting for specific materials, systems and methods

We require information from authors about some types of materials, experimental systems and methods used in many studies. Here, indicate whether each material, system or method listed is relevant to your study. If you are not sure if a list item applies to your research, read the appropriate section before selecting a response.

Materials & experimental systems

n/a	Involved in the study
☐	☒ Antibodies
☒	☐ Eukaryotic cell lines
☒	☐ Palaeontology and archaeology
☒	☐ Animals and other organisms
☒	☐ Clinical data
☒	☐ Dual use research of concern

Methods

n/a	Involved in the study
☒	☐ ChIP-seq
☒	☐ Flow cytometry
☒	☐ MRI-based neuroimaging

Antibodies

Antibodies used	Anti-GFP11-Invitrogen: PA5-109258<br>Anti-mCherry- CST: E5D8F<br>Anti-Rabbit IGG-HRP- CST: 7074
Validation	Anti-GFP11-Invitrogen: PA5-109258, validated through immune reaction with synthetic GFP11 peptide. Tested by manufacturer with GFP11 tagged antibody.<br>Anti-mCherry- CST: E5D8F. Validated by manufacturer using recombinant c-myc tagged mCherry protein.<br>Anti-Rabbit IGG-HRP- CST: 7074. Validated independently by 11557 unique citations for this antibody.
